# C20orf24 promotes colorectal cancer progression by recruiting Rin1 to activate Rab5‐mediated mitogen‑activated protein kinase/extracellular signal‐regulated kinase signalling

**DOI:** 10.1002/ctm2.796

**Published:** 2022-04-07

**Authors:** Yang Wang, Jing Zhang, Can‐Can Zheng, Zi‐Jia Huang, Wei‐Xia Zhang, Yun‐Lin Long, Gui‐Bin Gao, Yue Sun, Wen Wen Xu, Bin Li, Qing‐Yu He

**Affiliations:** ^1^ MOE Key Laboratory of Tumor Molecular Biology and Key Laboratory of Functional Protein Research of Guangdong Higher Education Institutes Institute of Life and Health Engineering College of Life Science and Technology Jinan University Guangzhou China; ^2^ Department of Radiology The First Affiliated Hospital Jinan University Guangzhou China; ^3^ MOE Key Laboratory of Tumor Molecular Biology and Guangdong Provincial Key Laboratory of Bioengineering Medicine National Engineering Research Center of Genetic Medicine Institute of Biomedicine College of Life Science and Technology Jinan University Guangzhou China

Dear Editor,

Epidermal growth factor receptor (EGFR)‐mediated mitogen‑activated protein kinase/extracellular signal‐regulated kinase (MAPK/ERK) signalling is highly activated in colorectal cancer (CRC).^1^, ^2^ Small GTPase Ras‐related protein Rab‐5A (Rab5) is a critical player in transducing this oncogenic signal.^3^, ^4^ Rab5 activation has been known to be enhanced by relevant guanine exchange factors (GEFs)^5^; however, the mechanism by which GEF activates Rab5 is poorly understood. The current work represents our effort in the comprehensive characterisation of a “dark” protein, C20orf24, which works as a Rab5 activator to promote colorectal tumorigenesis through EGFR/MEK/ERK signalling pathway.

Human Proteome Organization launched a project named neXt‐CP50, aiming to characterise those proteins with completely unknown functions, termed uncharacterised protein existence level 1 (uPE1) proteins.^6^ These “dark” proteins are a rich resource for exploring novel tumour‐associated proteins. Here, we screened for novel EGFR signalling regulators in CRC from 35 dedicated uPE1 proteins using the The Cancer Genome Atlas (TCGA) data set. C20orf24 was the top‐ranked protein upregulated in CRC tissues among the 35 uPEs (Figure 1A). C20orf24 co‐exists in a fusion gene TGIF2‐C20orf24, which constitutively occurs during read‐through transcription between the TGIF2 and C20orf24 genes (Figure S1A). We found that the copy numbers of the three genes were markedly upregulated in CRC tissues (Figure S1B), while the expression of C20orf24 was higher than that of TGIF2 or TGIF2‐C20orf24 in CRC tissues and cell lines (Figure S1C,D), as confirmed by quantitative real‐time polymerase chain reaction (qRT‐PCR) in 17 CRC tumours (Figure S1E). Interestingly, higher ability in cell proliferation was only observed in C20orf24‐overexpressing HCT116 cells (Figure S1F‐H), suggesting that C20orf24, but not TGIF2 or TGIF2‐C20orf24, may be a novel regulator for tumorigenesis. To confirm this, a tissue microarray consisting of 99 CRC tissues and Gene Expression Omnibus (GEO) data sets were analysed, showing that C20orf24 was significantly upregulated in the majority of CRC tissue samples (Figure 1B–E). Furthermore, high C20orf24 expression was positively correlated with shorter survival (Figure 1F,G), and significantly correlated with pathological N and M stages in 99 CRC patients (Table S1).

**FIGURE 1:**
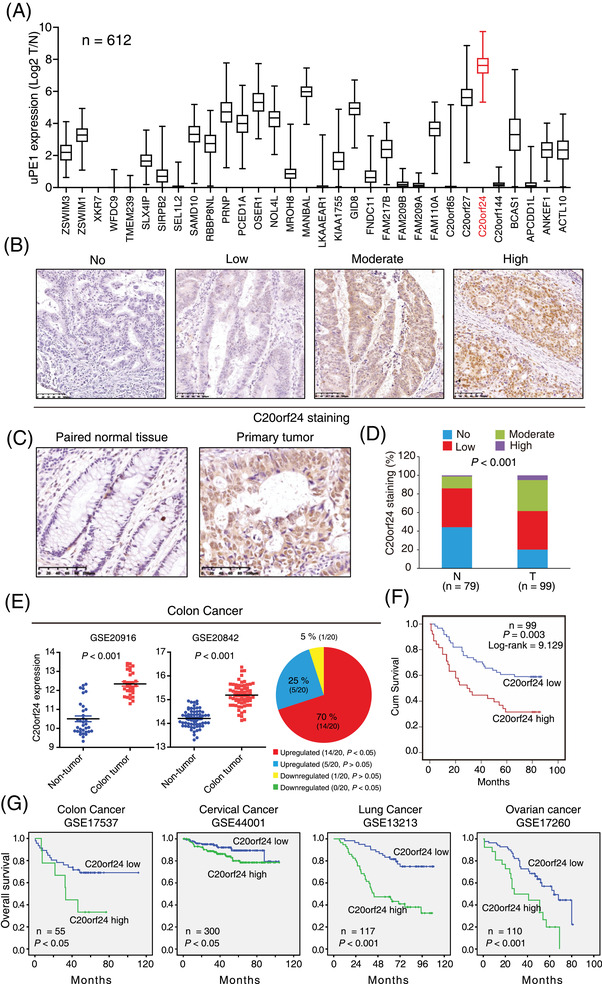
Chromosome 20 open reading frame 24 (C20orf24) in colorectal carcinoma (CRC) is correlated with poor prognosis of patients. (A) Analysis of expression level of 35 uncharacterised protein existence level 1 (uPE1) in CRC tissue using The Cancer Genome Atlas (TCGA) data sets (*n* = 612). (B) Representative images of CRC with immunohistochemical staining scores of no to high (0‐3) for C20orf24. (C) An example of C20orf24 in paired primary CRC and non‐tumour tissues. (D) Statistical analysis showed that C20orf24 expression was significantly increased in CRC tissues as compared with non‐tumour tissues. (E) Oncomine data sets were acquired for comparing the mRNA expression of C20orf24 in CRC tumours and non‐tumour tissues, and representative data were shown. (F) Kaplan‐Meier plots were used to compare the overall survival of 99 patients with CRC stratified according to the C20orf24 expression level. (G) Kaplan‐Meier plots based on Gene Expression Omnibus (GEO) data sets of patients with colon cancer (GSE17537), cervical cancer (GSE44001), lung cancer (GSE13213) and ovarian cancer (GSE17260). The survival curves showed that high C20orf24 expression is correlated with the poor prognosis of CRC patients

To study the role of C20orf24 in proliferation of CRC cells, overexpression and knockdown of C20orf24 were performed (Figure S2A,B). C20orf24 overexpression had a higher proliferation rate, while C20orf24 knockdown stably or transiently suppressed cell proliferation in vivo and in vitro (Figure 2A,B; Figure S2C‐E), showing a decrease in tumour volume and Ki‐67 staining (Figure 2C). Stable isotope labelling with amino acids in cell culture (SILAC)‐based proteomics (Figure S3A‐C; Figure 2D; Table S2) revealed that the C20orf24‐regulated proteins were mainly enriched in the MAPK/ERK signalling pathway (Figure 2E), as confirmed by positive correlation between C20orf24 and p‐MEK and p‐ERK expression (Figures 2F,G). Moreover, with pimasertib^7^ treatment, the cell growth, p‐MEK and p‐ERK in cells overexpressing C20orf24‐HA were reduced to the level in control (Figure S3D‐F).

**FIGURE 2:**
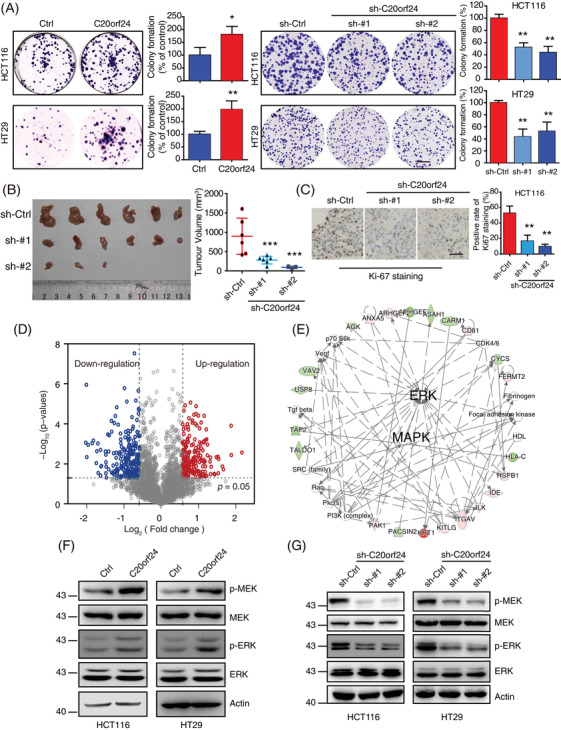
Chromosome 20 open reading frame 24 (C20orf24) exerts oncogenic activities in vivo and in vitro through the mitogen‑activated protein kinase/extracellular signal‐regulated kinase (MAPK/ERK) signalling pathway. (A) Colony formation assays were performed to determine the ability of HCT116 and HT29 cells to form colonies by manipulating C20orf24 expression. Scale bar, 5 mm. (B) Images of the tumours and growth curves of the subcutaneous tumours formed by C20orf24‐knockdown HCT116 cells (*n* = 6). (C) Immunostaining of Ki‐67 and quantification of the proliferation index in the tumours; scale bar, 50 μm. Bars, SD; *, *p* < .05; **, *p* < .01, ***, *p* < .001. (D, E) Stable isotope labelling with amino acids in cell culture (SILAC)‐based quantitative proteomics identified MAPK/ERK signalling pathways activated by C20orf24. (D) C20orf24‐regulated proteins using SILAC‐based proteomics. Differentially expressed proteins in C20orf24‐overexpression HCT116 cells are represented by a volcano plot. (E) Ingenuity Pathway Analysis (IPA) suggested that the MAPK/ERK pathways are involved in the functional role of C20orf24 in CRC progression. (F, G) The expression levels of p‐ERK and p‐MEK were compared in HCT116 and HT29 cells with C20orf24 overexpression or knockdown by Western blotting, and actin was used as a loading control. The experiment was performed in culture medium containing 2% serum. Bars, SD; *, *p* < .05; **, *p* < .01, ***, *p* < .001

We next performed immunoprecipitation coupled with mass spectrometry (Figure 3A and Table S3) to identify the binding partner of C20orf24, a direct interaction between C20orf24 and Rab5 was found and subsequently confirmed by immunoprecipitation exogenously and endogenously (Figure 3B,C). Interestingly, overexpression of C20orf24 increased the activity of Rab5 (GTP‐bound form) in CRC cells (Figure 3D). Rab5 deletion suppressed the CRC proliferation promoted by C20orf24 (Figure S4A,B). Induction of Rab5‐S34N (inactive form),^8^ but not wild‐type (WT) Rab5 or Rab5‐Q79L (active form), abrogated the C20orf24‐induced p‐MEK and p‐ERK expression (Figure 3E and S4C). The activation of Rab5 (Figure 3F), phosphorylation of EGFR and ERK and degradation of EGFR (Figure S4D) were markedly decreased in C20orf24‐knockdown cells after epidermal growth factor (EGF) stimulation. Pulse‐chase assay showed a relatively rapid decrease in p‐EGFR and p‐ERK expression in C20orf24‐knockdown CRC cells upon exposure to EGF (Figure S4E,F), suggesting that C20orf24 is essential for Rab5 activation, contributing to EGFR/MEK/ERK signalling.

**FIGURE 3:**
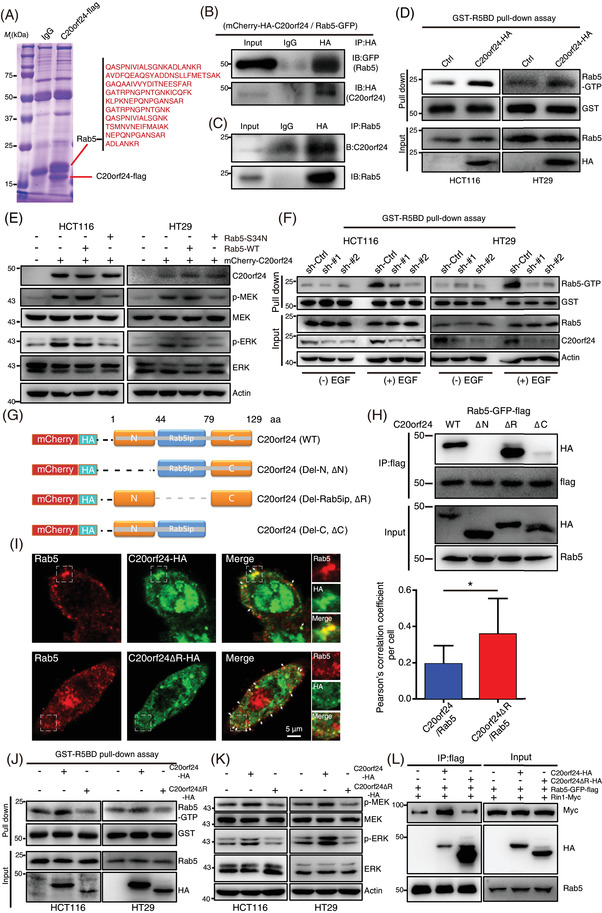
Interaction of chromosome 20 open reading frame 24 (C20orf24) and Ras‐related protein Rab‐5A (Rab5) activates mitogen‑activated protein kinase/extracellular signal‐regulated kinase (MAPK/ERK) signalling. (A) Coomassie blue staining showing the C20orf24‐associated proteins in HCT116 cells. The peptides for Rab5 identified by mass spectrometry (MS) were labeled in red. (B) HCT116 cells transfected with mCherry‐HA‐C20orf24 and Rab5‐GFP plasmids were subjected to co‐immunoprecipitation (Co‐IP) assays. (C) The endogenous interaction between C20orf24 and Rab5 was confirmed by immunoprecipitation. (D) HCT116 and HT29 with or without the overexpression of C20orf24 were compared for Rab5 activity by using GST‐R5BD pull‐down assay. (E) C20orf24‐overexpressing plasmids were transfected into HCT116 and HT29 cells together with the plasmid expressing Rab5‐WT or Rab5‐S34N as indicated; additionally, the expression levels of p‐MEK, MEK, p‐ERK and ERK were detected by Western blotting. (F) C20orf24‐depleted HCT116 and HT29 cells with or without the presence of epidermal growth factor (EGF) stimulation were determined for Rab5 activity by using GST‐R5BD pull‐down assay. (G) Schematic diagram of the domains of C20orf24 and mutation design for N‐terminal deletion (Del‐N, ΔN), Rab5ip domain deletion (ΔR) and C‐terminal deletion (Del‐C, ΔC). The blue line represents the Rab5ip domain. (H) The interaction of wild‐type or mutant C20orf24 with Rab5 was detected by co‐immunoprecipitation assay. (I) The HCT116 cells expressing C20orf24‐HA or C20orf24ΔR‐HA were subjected to immunostaining for endogenous Rab5, and imaged by confocal microscopy. The overlaps of Rab5 with C20orf24 or C20orf24ΔR were indicated by white arrows, and their correlations per cell were quantified. Data are represented as mean ± SD. *n* = 5‐10 cells. (J, K) HCT116 and HT29 cells were transfected with C20orf24 or C20orf24ΔR plasmids, the Rab5 activity was detected by using GST‐R5BD pull‐down assay (J), and the expression levels of p‐MEK, MEK, p‐ERK and ERK were compared by Western blotting (K). (L) HCT116 cells expressing Rab5‐GFP‐flag and Rin1‐myc were transfected with C20orf24 or C20orf24ΔR, their effects on the interaction of Rab5 and Rin1 was compared by a co‐immunoprecipitation assay. MEK, mitogen‐activated protein kinase kinase

A comparison of Rab5ip amino acid sequences in different species indicated that the 44‐79 amino acids of Rab5ip domain are highly conserved (Figure S5A). Therefore, we generated three C20orf24‐truncated mutants, including N‐terminal deletion (ΔN), Rab5ip domain deletion (ΔR) and C‐terminal deletion (ΔC), for immunoprecipitation assays in CRC cells (Figure 3G). Unexpectedly, Rab5 was bound to the C20orf24 mutant with a Rab5ip domain deletion (ΔR), but not to the mutants with N‐ or C‐terminal deletion (Figure 3H). C20orf24‐formed punctate dots were partially co‐localised with endogenous Rab5, ΔR showed higher overlap with Rab5, while ΔN and ΔC were diffuse in cell (Figure 3I and S5B). C20orf24ΔR neither enhanced the activity of Rab5 (Figure 3J) nor increased the phosphorylated MEK and ERK (Figure 3K). These results illustrate that the N‐ and C‐terminal fragments of C20orf24, but not the Rab5ip domain, are required for its association with Rab5.

Ras and Rab interactor 1 (Rin1) is a specific GEF that enhances Rab5 activity and induces Ras/MEK/ERK signalling.^9^ We found that C20orf24‐WT, but not C20orf24ΔR, enhanced the interaction between Rab5 and Rin1, indicating that the Rab5ip domain of C20orf24 is essential for Rab5 to recruit Rin1 (Figure 3L). Interestingly, an interaction between C20orf24 and Rin1 was observed, which could be reduced when Rab5ip domain was deleted (Figure S5C), suggesting that Rab5ip domain of C20orf24 is critical for its binding to Rin1. Moreover, we observed that C20orf24 exhibited stronger interaction with Rab5‐S34N than Rab5‐Q79L (Figure S5D). This suggests that upon binding with Rin1, C20orf24 exhibited higher affinity with the GDP status of Rab5, which is required for Rab5 activation. We next found that C20orf24, but not C20orf24ΔR, decreased the association of Ras with Rin1, thereby enhancing the interaction between Ras and Raf to activate Raf signalling,^10^ as indicated by the increased phosphorylation of Raf (Figure S5E). The release of Ras from Rin1 was also evidenced by Western blot analysis (Figure S5F). In vivo and in vitro studies showed that the effects of C20orf24 on cell proliferation and tumour growth could be reduced by Rab5‐S34N and Rab5ip domain deletion (Figure 4A–C). In conclusion, we show that N‐ and C‐terminal fragments of C20orf24 are essential for its binding with Rab5, while the Rab5ip domain of C20orf24 is responsible for its binding to Rin1. Through such interactions, C20orf24 recruits Rin1 to enhance Rab5 activity and release Ras from Rin1‐Ras complex to activate MEK/ERK signalling, contributing to the promotion of CRC development (Figure 4D). Our findings demonstrate that C20orf24 is an important oncoprotein that serves as a potential biomarker and therapeutic target for CRC.

**FIGURE 4:**
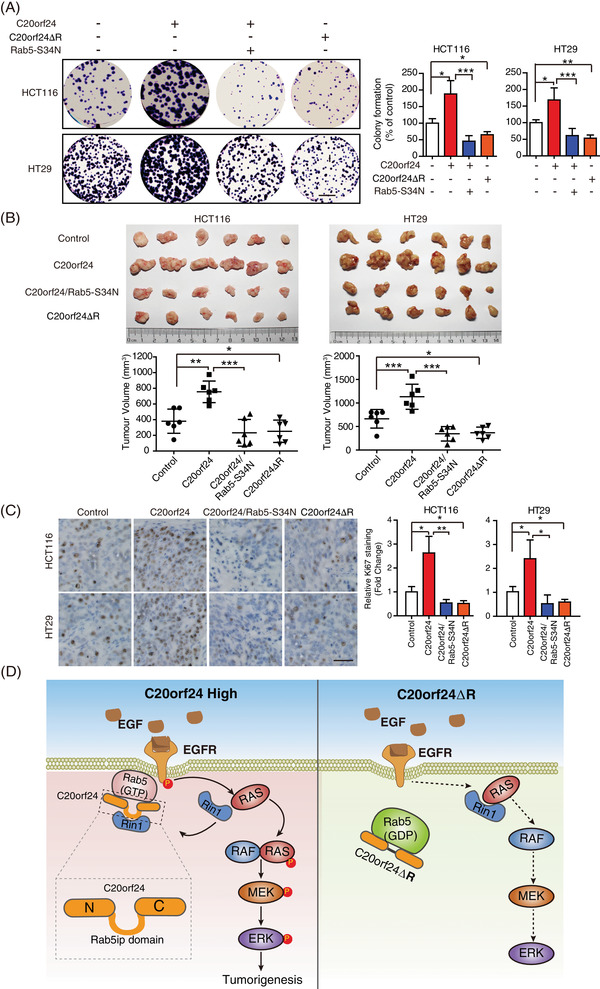
C20orf24ΔR functions as a tumour suppressor in vivo and in vitro. (A) Colony‐formation assay showing the inhibitory effect of Rab5‐S34N and C20orf24ΔR on HCT116 and HT29 cell proliferation. Note that Rab5‐S34N blocked the cell proliferation enhanced by chromosome 20 open reading frame 24 (C20orf24) and C20orf24ΔR decreased the growth of both colorectal carcinoma (CRC) cells; Scale bar, 5 mm. (B) HCT116 and HT29 expressing indicated proteins were subjected to subcutaneous xenograft mice model. The photograph represents excised tumours from the four groups (*n* = 6 per group). The tumour volumes are summarised in the line chart below. (C) The quantification of Ki‐67 proliferation index in the tumours; Scale bar, 50 μm. Bars, SD; *, *p* < .05; **, *p* < .01; ***, *p* < .001. (D) Schematic illustration of the mechanism of activation of Rab5 by C20orf24 to promote MEK/ERK signalling and CRC progression

## CONFLICT OF INTERESTS

The authors declare that they have no competing interests.

## Supporting information

SUPPORTING INFORMATION
