# Supplementary material for: C20orf24 promotes colorectal cancer progression by recruiting Rin1 to activate Rab5‐mediated mitogen‑activated protein kinase/extracellular signal‐regulated kinase signalling
Source: Clin Transl Med. 2022 Apr 7;12(4):e796. doi: 10.1002/ctm2.796 (PMC8989078; doi:10.1002/ctm2.796)
Supplement: Supplementary file 1 — SUPPORTING INFORMATION [file CTM2-12-e796-s001.pdf]

SUPPORTING INFORMATION FOR

C20orf24 promotes colorectal cancer progression by recruiting Rin1 to activate Rab5-mediated MAPK/ERK signaling

Yang Wang[1, #, *], Jing Zhang[1, 2, #], Can-Can Zheng[1, #], Zi-Jia Huang[1], Wei-Xia Zhang[1], Yun-Lin Long[1], Gui-Bin Gao[1], Yue Sun[1], Wen Wen Xu[3], Bin Li[1,*], Qing-Yu He[1,*]

MATERIALS AND METHODS

Reagent and resource

Antibodies, biological samples, chemicals and purified proteins, commercial assays, cell lines, animal models, oligonucleotides, recombinant plasmids, software and algorithms are listed in the supplementary Table S4.

Cell culture condition

Colorectal cancer cell lines HCT116 and HT29 were maintained in RPMI-1640 (Life Technologies, Gaithersburg, MD, USA) supplemented with 10% fetal bovine serum (FBS, Life Technologies) at 37 ˚C in 5 % CO_2.

Colony formation assay

Cells were seeded in 6-well or 12-well plates at a density of 400 cells/well. After 15 days, the cells were washed twice with PBS, and then fixed with methanol for 30 min and stained with 1% crystal violet for 10 min at room temperature. The colonies were then counted.

WST-1 assay, qRT-PCR and Western blotting

The cell viability was determined by using WST-1 assay (Beyotime, Jiangsu, China) and qRT-PCR were performed as described previously [1,2]. For Western blotting, cells were lysed in lysis buffer (Cell Signaling Technologies, Danvers, MA, USA) according to manufacturer's instructions, and protein concentration was determined with a BCA kit (Thermo Fisher Scientific, Waltham, MA, USA). Protein samples were loaded into sodium dodecyl sulfate (SDS)-PAGE gel for electrophoresis and transferred to PVDF membrane (Millipore, Bedford, MA, USA). After blocking with 5% fat-free milk for 1 h, the membranes were incubated with indicated primary antibodies overnight at 4˚C, and then incubated with the corresponding secondary antibodies at room temperature. The signals were detected with Clarity ECL Western Blot Substrate kit (Bio-Rad, Hercules, CA, USA).

Immunofluorescence

Cells were transfected with indicated plasmids (1 μg) for 24 h, fixed in 4% paraformaldehyde and permeabilized with 0.1% TrintonX-100 for 10 min before being blocked with 10% BSA for 2 h. The cells were then stained with indicated antibodies according to manufacturer's instructions. The cells were washed 3 times with 1% TBST, and followed by laser scanning confocal microscopy observation (Carl Zeiss AG, Jena, Germany).

In-solution trypsin digestion, LC-MS/MS analysis and bioinformatics analysis

Protein digestion and MS analysis were performed as described previously [3]. In detail, protein lysates were subjected to reduction, alkylation and then loaded into an ultracentrifuge

filter (30-kDa; Sartorius Stedim Biotech, Shanghai, China), followed by centrifugations with 8 M urea and five-times volume of 50 mM NH_4HCO_3, respectively. After further trypsin digestion overnight, the peptide samples were vacuum-freeze-dried and resuspended in anhydrous acetonitrile solution for further desalination with a Mono-TIPTM C18 Pipette Tip (GL Sciences, Tokyo, Japan). The eluted peptides were analyzed in an Orbitrap Fusion Lumos mass spectrometer (Thermo Fisher Scientific), and the raw data were searched using Proteome Discoverer software (Thermo Fisher Scientific). An FDR of 1% was set to identify proteins. The differentially expressed proteins were analyzed by Ingenuity Pathway Analysis (IPA, Ingenuity Systems, Redwood City, CA, USA) as described previously [1].

Tissue microarray and immunohistochemistry (IHC)

IHC was performed as described previously [4]. A human colorectal tumor tissue microarray containing 99 cases of human CRC tissues and 79 cases of adjacent normal tissues with survival data (Outdo Biotech, Shanghai, China) was deparaffinized in xylene, rehydrated in a graded series of ethanol solutions and processed for IHC. After antigen retrieval, the slides were incubated with C20orf24 antibody overnight at 4°C, washed with PBS, and then incubated with the appropriate peroxidase-conjugated secondary antibody (Dako, Diagnostics, Mississauga, ON, USA). Immunostaining was visualized using 3,3’-diaminobenzidine (Dako), which served as a chromogen, and the sections were counterstained with hematoxylin. C20orf24 immunostaining was evaluated based on the staining intensities which were classified into the following four categories: 0, no staining; 1, low staining; 2, moderate staining; 3, high staining.

Tumorigenicity in nude mice

BALB/c nude mice aged 4-6 weeks were purchased from Model Animal Research Center of Nanjing University (Nanjing, Jiangsu, China). Tumor xenograft experiments were performed according to our previous study [4,5]. Briefly, cells were inoculated subcutaneously on the flanks of nude mice (n ≥ 6 per group). We calculated the tumor volume according to the formula $V = 1/2\ ab^2$, with "a" representing the longest diameter and "b" representing the shortest diameter. All animal experiments were performed according to the guidelines for the care and use of laboratory animals and were approved by The Ethics Committee for Animal Experiments of Jinan University (Approval number: 20190312-01).

GST-R5BD pull-down assay

The GST-R5BD fusion protein pull-down assay is a conventional method to detect the activity of Rab5 (GTP-Rab5). The assay was based on the specific binding of Rab5-GTP by the R5BD of Rabaptin5 (a Rab5 effector). GST-Rab5 binding domain (R5BD) pull-down assay was performed as previously described[6]. In brief, cell lysates were incubated with 3 μg of GST or GST-R5BD bound to the glutathione-Sepharose 4B beads (Beyotime) 5-10 min at 4°C under rotation. The beads were subsequently rinsed with lysis buffer, resuspended in RIPA sample buffer (Cell Signaling Technologies), boiled for 5 min, and then subjected to SDS-PAGE and Western blotting.

Co-immunoprecipitation (Co-IP) and mass spectrometry (MS)

Whole cell lysates from HCT-116 cells expressing C20orf24-flag were prepared using lysis buffer (Beyotime). Co-IP was performed using indicated antibodies, and the immune complexes were captured on Protein A/G agarose beads (Santa Cruz, CA, USA), and subjected to Western blotting analysis.

For MS analysis, the C20orf24-flag complexes were separated and the gels were stained with Coomassie brilliant blue. The gel bands were excised and subjected to in-gel digestion. The isolated peptides were dissolved in buffer containing 0.1% formic acid and 2% acetonitrile and analyzed using Orbitrap Fusion Lumos mass spectrometer. The RAW files were subjected to Proteome Discoverer 2.1 Software for protein identification according to the Uniprot human protein database (released in Dec 2019). The following search criteria were employed: The proteins were digested by trypsin; two missed cleavages were allowed; carbamidomethylation was set as fixed modification, whereas oxidation (M) was considered as variable modifications; initial mass deviation of precursor ion and fragment ions were allowed up to 30 ppm and 0.1 Da, respectively; FDR is 1%.

Analysis of gene expression and survival data from cancer patient datasets

The copy number, gene expression of C20orf24, TGIF2, TGIF2-C20orf24 and uPEs in CRC patients and CRC cell lines were analyzed in the datasets from The Cancer Genome Atlas (TCGA) and Cancer Cell Line Encyclopedia (CCLE) with Ordino (https://ordino.caleydoapp.org), a web-based analysis tool[7]. C20orf24 expression in CRC patient microarray datasets were analyzed by Oncomine (https://www.oncomine.org/resource/login.html). The datasets with survival data from

patients with colon cancer [8], cervical cancer [9], lung cancer [10], and ovarian cancer [11] were downloaded from the GEO database (accession numbers GSE17537, GSE44001, GSE13213, GSE17260). Gene expressions were further divided into high and low levels using median expression level as the cutoff point for Kaplan-Meier survival analyses.

Statistical analysis

The data were expressed as the mean ± SD and compared using ANOVA. The expression level of C20orf24 in tumor was compared with that in non-tumor tissues using paired or unpaired *t*-test. The correlation between C20orf24 and clinicopathological parameters was assessed using Fisher exact test. For confocal analysis, the overlap of red and green was statistically analyzed by Pearson's Correlation coefficient. Survival analysis was performed by Kaplan-Meier method with the log-rank test. All *in vitro* experiments were repeated at least three times. All the data were analyzed by two-tailed unpaired Student's *t*-test using GraphPad Prism software. $P < 0.05$ was considered to be statistically significant.

Discussion

This study characterized a novel oncoprotein, C20orf24, which is a previously functionally unknown gene located in the 20q11.23 region of Chromosome 20. C20orf24 promotes CRC tumorigenesis by taking a significant part in regulating canonical EGFR/MEK/ERK signaling. As illustrated in Figure 4D, in the process of the signal transduction, the N- and C-terminal domains of C20orf24 are essential for its interaction with Rab5, while the Rab5ip domain of C20orf24 is responsible for its binding to Rin1. By means of such interactions, C20orf24

recruits Rin1 to enhance Rab5 activity and releases Ras from Rin1-Ras complex to activate MEK/ERK signaling, contributing to the promotion of CRC development. Our findings demonstrate that C20orf24 is an important oncoprotein, serving as a potential biomarker and therapeutic target for CRC.

The entire human genome contains 20352 protein-coding genes in which 15428 (76%) have been validated at the protein level according to the neXtProt project released by the Human Proteome Organization. Among these identified proteins, there is a panel of molecules (~1260) with completely unknown function [12]. As one of our efforts in C-HPP projects, we focused on the functionally uncharacterized proteins in 20q11.23 region, in which C20orf24 gene has aberrant expression in human cancer [13]. Here, we found that TGIF2-C20orf24 locus represents naturally occurring read-through transcription between the neighboring TGIF2 and C20orf24 genes. A series of gain- and loss-of-function experiments and functional assays demonstrated that only C20orf24, but not TGIF2 or TGIF2-C20orf24, could promote CRC cell proliferation and motility *in vivo* and *in vitro*. In addition, C20orf24 was found upregulated in CRC tumor tissues, and its expression was associated with patient survival, implicating C20orf24 to be a functional molecule in human cancer. Our following-up proteomic analysis suggested that C20orf24 probably participates in CRC progression by involving MEK/ERK signaling. This involvement was then confirmed by the validation experiment using pimasertib, a highly selective small-molecule inhibitor of the protein kinase MEK1/2, which blocked the oncogenic phenotypes induced by C20orf24, including cell proliferation, colony formation, invasion and migration, verifying that C20orf24 may be a novel regulator in mediating MEK/ERK signaling pathways.

EGFR/MAPK signaling pathway regulates a wide spectrum of cellular functions including proliferation, survival and invasion [14]. Genetic abnormality of the EGFR/MAPK pathway participates in the development of multiple types of cancer, and remains the main challenge for CRC treatment with the new developed therapeutics [15,16]. Activation of Rab5 plays an important role in cancer progression through regulating EMT and the ERK/MMP2 signaling pathway [17,18]. However, the mechanism in the regulation of Rab5 activity remains to be elucidated. In this study, we unexpectedly identified C20orf24 as a binding partner of Rab5 by using IP-mass spectrometry, and further demonstrated that C20orf24 induces Rab5 activation to promote cancer development. These results inspired us to speculate that C20orf24 may play an important role in the EGFR/MAPK/ERK pathway. It has been documented that EGFR upon ligand binding activates Rab5 and undergoes rapid activation [19]. Rab5-S34N, a dominant negative mutant of Rab5, represses EGF-stimulated cell proliferation by inhibiting EGFR/MAPK/ERK signaling [20,21], while EGFR signal can be potentiated by overexpression of Rab5-Q79L, an activated form of Rab5. These observations suggest that Rab5 activation induced by C20orf24 contributes to EGFR/MAPK/ERK signaling-mediated oncogenic activity.

We then determined that C20orf24 directly interacts with Rab5 to boost MEK/ERK signaling through the N- and C-terminals but not the Rab5ip domain (44-79 aa) of C20orf24. This is surprising but interesting because that Rab5ip domain in C20orf24 was predicted to bind with Rab5, as recorded in Pfam database (PF07019). In fact, we found that this domain is not required for Rab5 binding, as evidenced by that deletion of Rab5ip domain enhanced the interaction between truncated C20orf24 and Rab5. According to LocSigDB, a database of

protein localization signals [22], three potential endosome/ lysosome-located signals (YXX [VILWCM]) were predicted in C20orf24: YLYF (83-86aa), YLQI (89-92aa) and YTAI (123-126aa). These three motifs are not located within Rab5ip domain (44-79 aa), supporting our observations that C20orf24 is located in endosome/lysosome, while deletion of Rab5ip domain did not abolish such a cellular localization (Figure 3I). Functionally, knockdown of C20orf24 not only diminished EGF-induced activation of EGFR/MEK/ERK pathway, but also inhibited tumor growth *in vivo* and *in vitro*. Moreover, the inactivated form of Rab5, Rab5-S34N, could significantly abolish the oncogenic effect of C20orf24. These results demonstrate that C20orf24 acts as a novel activator of Rab5 to promote CRC tumorigenesis *via* EGFR/MAPK/ERK signaling.

On the other hand, we uncovered that C20orf24 does use its Rab5ip domain to recruit Rin1, a canonical GEF of Rab5 [23], to activate Rab5, as supported by the evidences that deletion of Rab5ip domain could effectively decrease the binding of C20orf24 to Rin1 in CRC cells. It has been reported that Rin1 can trap activated Raf and Ras for inhibiting MAPK signaling pathway [24]. Here, we found that the competitive binding of C20orf24 with Rin1 can release Ras from the sequestration of Rin1, subsequently activating Ras/MAPK signaling. Ras inhibitors did not achieve satisfactory results in clinical trials probably due to the fact that these inhibitors are not able to hit specific target proteins [25]. Here, we identified C20orf24 as a novel activator of Ras/MAPK to promote CRC tumorigenesis, suggesting that C20orf24 may be a new target for cancer treatment, providing another therapeutic option for CRC patients with RAS/MAPK aberrations.

Based on these observations, we testified that an artificial protein, C20orf24ΔR with the

deletion of Rab5ip domain could effectively decrease the binding of C20orf24 with Rin1 in CRC cells. Our results from a series of functional assays illustrated that the artificial C20orf24ΔR markedly inhibited Rab5 activity and MAPK/ERK signaling, therefore suppressing the growth of CRC cells and tumors *in vivo* and *in vitro*, suggesting that C20orf24ΔR can be developed as a promising chemotherapeutic agent for clinic application.

References

1. Wang Y, Zhang J, Huang ZH, et al. Isodeoxyelephantopin induces protective autophagy in lung cancer cells via Nrf2-p62-keap1 feedback loop. *Cell Death Dis.* 2017;8(6):e2876.
2. Li B, Tsao SW, Chan KW, et al. Id1-induced IGF-II and its autocrine/endocrine promotion of esophageal cancer progression and chemoresistance--implications for IGF-II and IGF-IR-targeted therapy. *Clin Cancer Res.* 2014;20(10):2651-2662.
3. Zhang J, Wang Y, Zhou Y, He QY. Jolkinolide B induces apoptosis of colorectal carcinoma through ROS-ER stress-Ca(2+)-mitochondria dependent pathway. *Oncotarget.* 2017;8(53):91223-91237.
4. Xu WW, Li B, Guan XY, et al. Cancer cell-secreted IGF2 instigates fibroblasts and bone marrow-derived vascular progenitor cells to promote cancer progression. *Nat Commun.* 2017;8:14399.
5. Zhang J, Zhou Y, Li N, et al. Curcumol Overcomes TRAIL Resistance of Non-Small Cell Lung Cancer by Targeting NRH:Quinone Oxidoreductase 2 (NQO2). *Adv Sci (Weinh).* 2020;7(22):2002306.
6. Zhou X, Xie S, Wu S, et al. Golgi phosphoprotein 3 promotes glioma progression via inhibiting Rab5-mediated endocytosis and degradation of epidermal growth factor receptor. *Neuro Oncol.* 2017;19(12):1628-1639.
7. Streit M, Gratzl S, Stitz H, Wernitznig A, Zichner T, Haslinger C. Ordino: a visual cancer analysis tool for ranking and exploring genes, cell lines and tissue samples. *Bioinformatics.* 2019;35(17):3140-3142.
8. Smith JJ, Deane NG, Wu F, et al. Experimentally derived metastasis gene expression profile predicts recurrence and death in patients with colon cancer. *Gastroenterology.* 2010;138(3):958-968.
9. Lee YY, Kim TJ, Kim JY, et al. Genetic profiling to predict recurrence of early cervical cancer. *Gynecol Oncol.* 2013;131(3):650-654.
10. Tomida S, Takeuchi T, Shimada Y, et al. Relapse-related molecular signature in lung adenocarcinomas identifies patients with dismal prognosis. *J Clin Oncol.* 2009;27(17):2793-2799.
11. Yoshihara K, Tajima A, Yahata T, et al. Gene expression profile for predicting survival in advanced-stage serous ovarian cancer across two independent datasets. *PLoS One.* 2010;5(3):e9615.
12. Paik YK, Lane L, Kawamura T, et al. Launching the C-HPP neXt-CP50 Pilot Project for Functional Characterization of Identified Proteins with No Known Function. *J Proteome Res.* 2018;17(12):4042-4050.
13. Wang Q, Wen B, Yan G, et al. Qualitative and quantitative expression status of the human chromosome 20 genes in cancer tissues and the representative cell lines. *J Proteome Res.* 2013;12(1):151-161.
14. Burotto M, Chiou VL, Lee JM, Kohn EC. The MAPK pathway across different malignancies: a new perspective. *Cancer.* 2014;120(22):3446-3456.

15. Doebele RC. Acquired Resistance Is Oncogene and Drug Agnostic. *Cancer Cell.* 2019;36(4):347-349.
16. Santarpia L, Lippman SM, El-Naggar AK. Targeting the MAPK-RAS-RAF signaling pathway in cancer therapy. *Expert Opin Ther Targets.* 2012;16(1):103-119.
17. Zhang D, Lu C, Ai H. Rab5a is overexpressed in oral cancer and promotes invasion through ERK/MMP signaling. *Mol Med Rep.* 2017;16(4):4569-4576.
18. Igarashi T, Araki K, Yokobori T, et al. Association of RAB5 overexpression in pancreatic cancer with cancer progression and poor prognosis via E-cadherin suppression. *Oncotarget.* 2017;8(7):12290-12300.
19. Chen PI, Kong C, Su X, Stahl PD. Rab5 isoforms differentially regulate the trafficking and degradation of epidermal growth factor receptors. *J Biol Chem.* 2009;284(44):30328-30338.
20. Barbieri MA, Fernandez-Pol S, Hunker C, Horazdovsky BH, Stahl PD. Role of rab5 in EGF receptor-mediated signal transduction. *Eur J Cell Biol.* 2004;83(6):305-314.
21. Zhang J, Sun Y, Zhong LY, et al. Structure-based discovery of neoandrographolide as a novel inhibitor of Rab5 to suppress cancer growth. *Comput Struct Biotechnol J.* 2020;18:3936-3946.
22. Negi S, Pandey S, Srinivasan SM, Mohammed A, Guda C. LocSigDB: a database of protein localization signals. *Database (Oxford).* 2015;2015.
23. Balaji K, Mooser C, Janson CM, Bliss JM, Hojjat H, Colicelli J. RIN1 orchestrates the activation of RAB5 GTPases and ABL tyrosine kinases to determine the fate of EGFR. *J Cell Sci.* 2012;125(Pt 23):5887-5896.
24. Han L, Colicelli J. A human protein selected for interference with Ras function interacts directly with Ras and competes with Raf1. *Mol Cell Biol.* 1995;15(3):1318-1323.
25. Degirmenci U, Wang M, Hu J. Targeting Aberrant RAS/RAF/MEK/ERK Signaling for Cancer Therapy. *Cells.* 2020;9(1).

Supplementary Figures

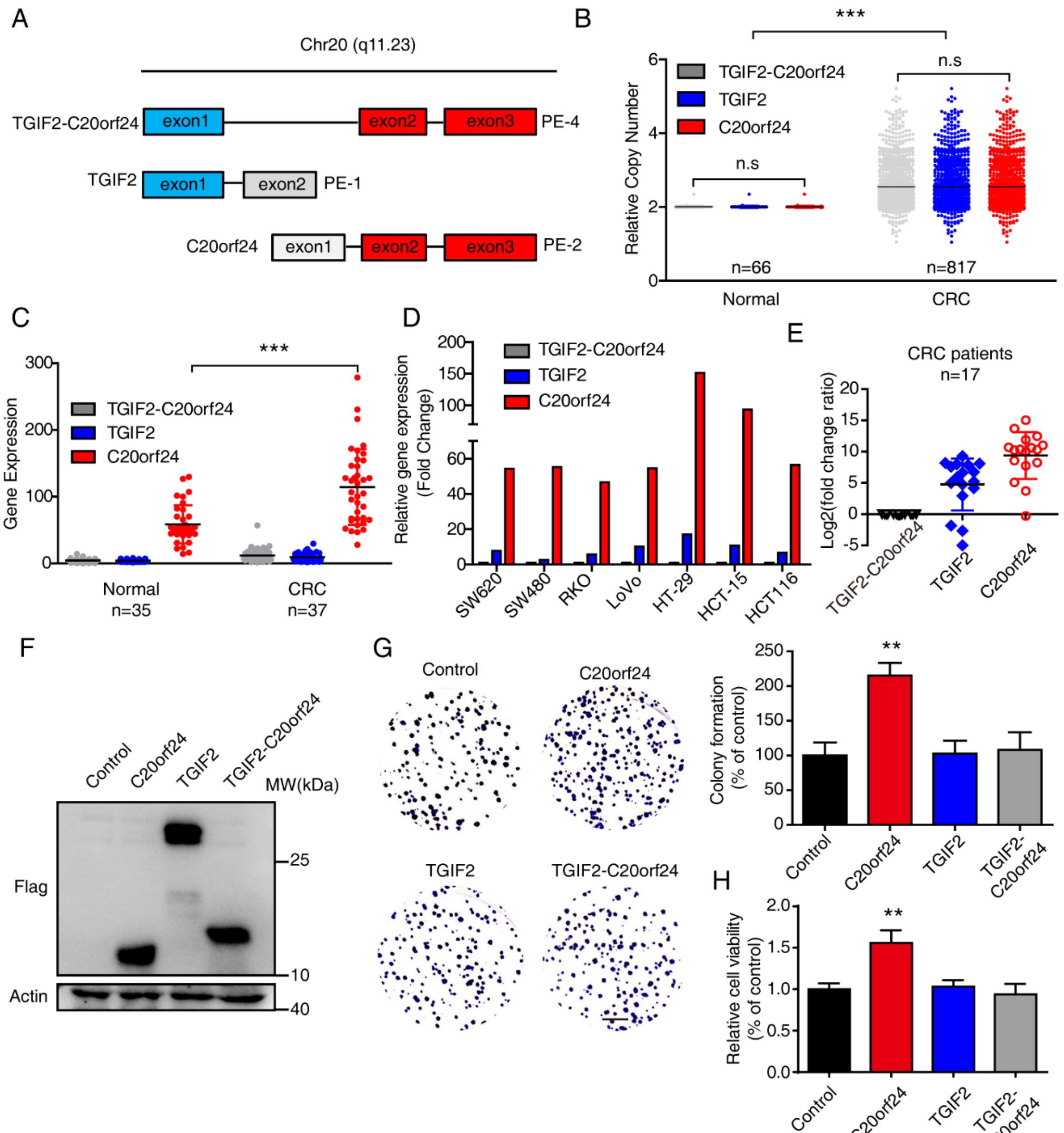

Figure S1

Figure S1. C20orf24, but not TGIF2 and TGIF2-C20orf24, is upregulated in CRC. (A) Gene locus of C20orf24, TGIF2 and TGIF2-C20orf24 in chromosome 20 (q11.23). **(B-D)** Comparison of copy number **(B)**, expression levels of C20orf24, TGIF2 and TGIF2-C20orf24 in CRC tumors and normal tissues **(C)**, as well as in various CRC cell lines **(D)**, using Ordino software. **(E)** The qRT-PCR was used to determine the mRNA expression of C20orf24,

TGIF2 and TGIF2-C20orf24 in 17 pairs of CRC tumors (T) and adjacent normal tissues (N). Their fold change of T/N ratios were plotted. **(F)** HCT116 sublines stably expressing flag-tagged C20orf24, TGIF2 or TGIF2-C20orf24, respectively, were screened out by G418, and their protein level was detected by Western blotting. The effect of C20orf24, TGIF2 and TGIF2-C20orf24 on proliferation of HCT116 cells was compared by using colony formation **(G)** and WST-1 assays **(H)**. Scale bar, 5 mm. Bars, SD; *, $P < 0.05$; ***, $P < 0.001$.

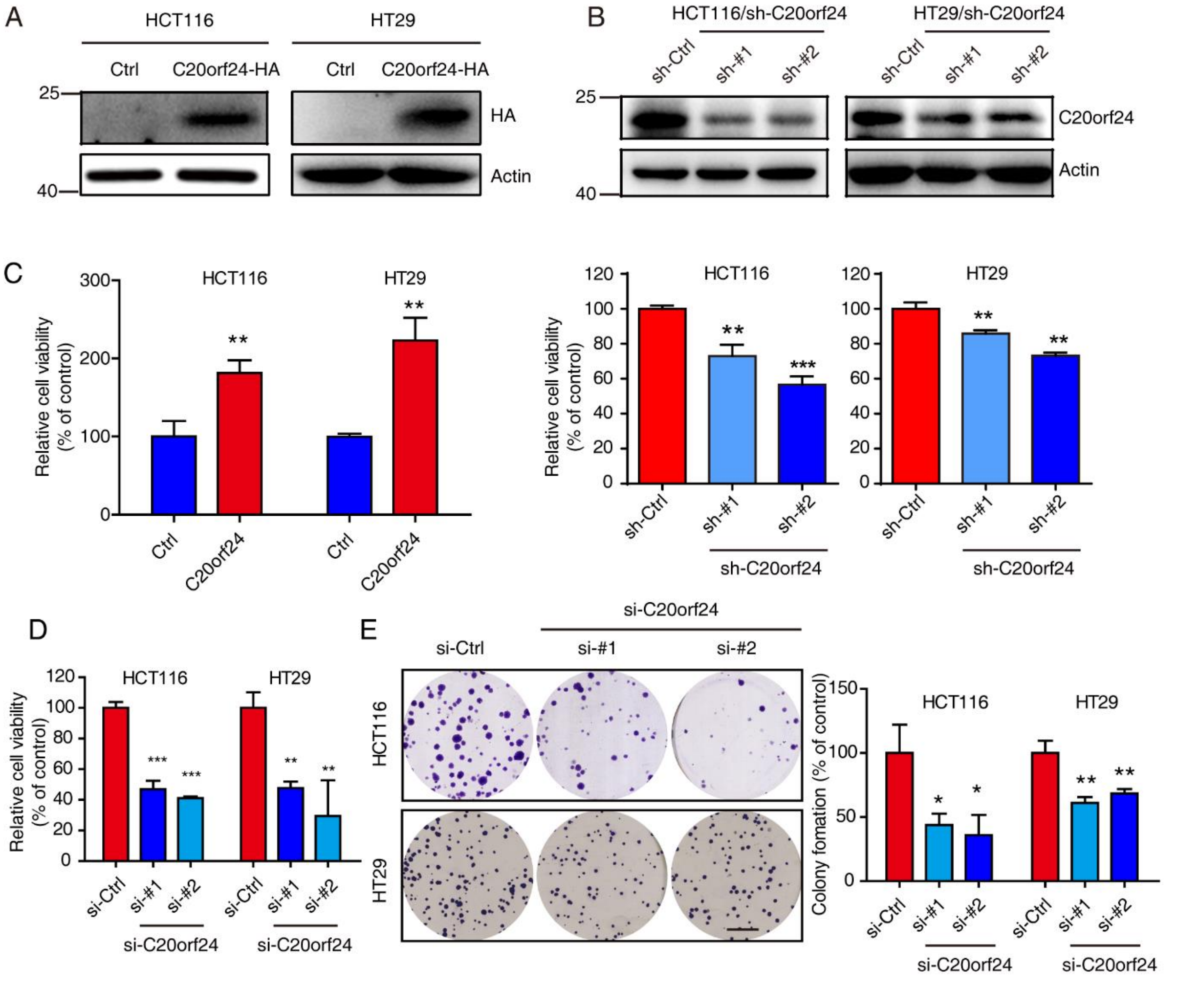

Figure S2

Figure S2. Role of C20orf24 on cell proliferation in CRC cells. (A, B) Successful stable overexpression **(A)** and knockdown **(B)** of C20orf24 in HCT116 and HT29 cells. **(C)** WST-1 assays were performed to determine the abilities to proliferate in HCT116 and HT29 cells with manipulation of C20orf24 expression. **(D, E)** Inhibitory effect of C20orf24 knockdown on cell proliferation. WST-1 and colony formation assays were performed to determine the abilities to proliferate **(D)** and form colonies **(E)** in HCT116 and HT29 cells with transiently transfection of two siRNAs against C20orf24. Scale bar, 5 mm. Bars, SD; *, $P < 0.05$; **, $P < 0.01$; ***, $P < 0.001$.

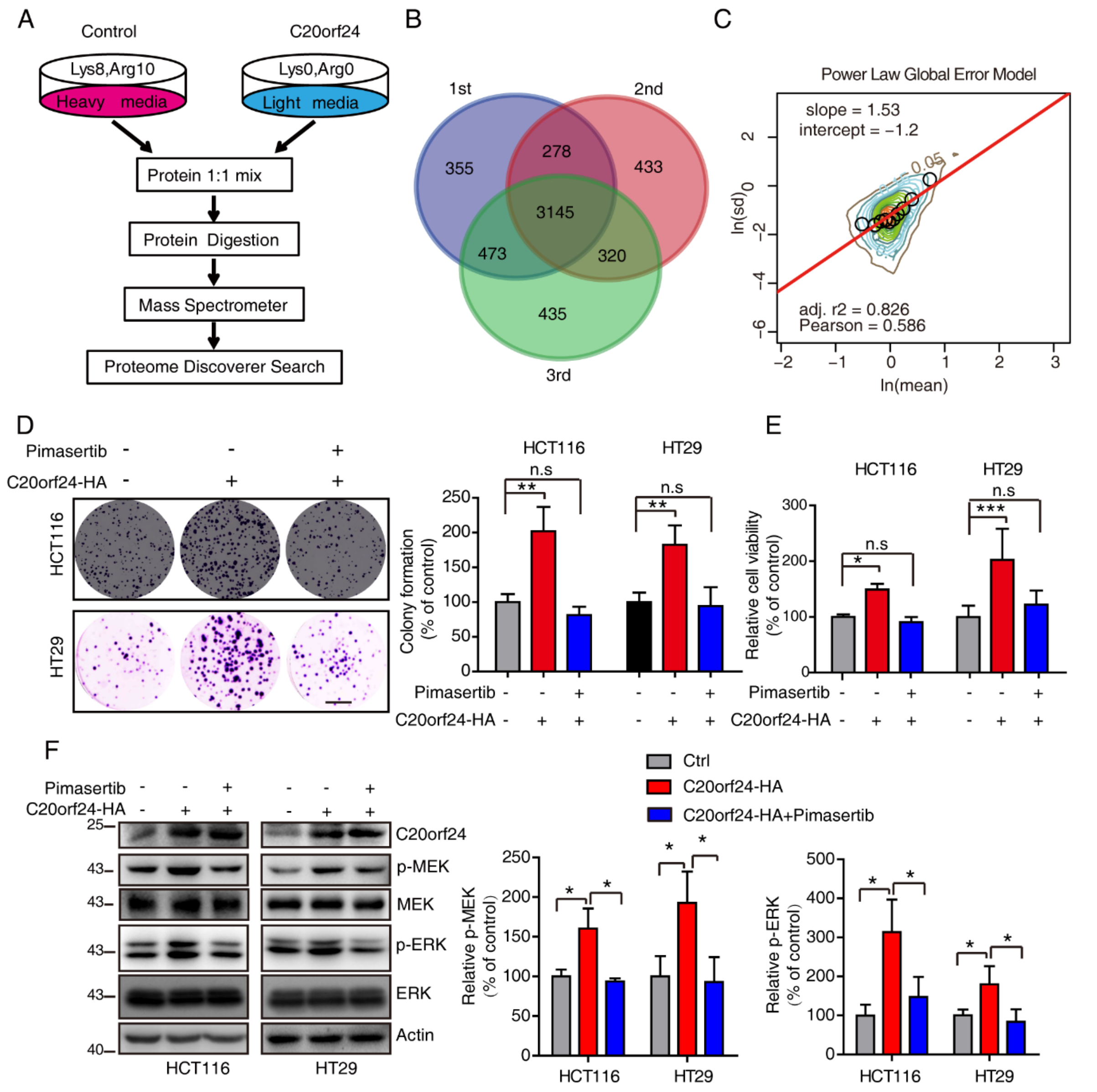

Figure S3

Figure S3. MEK/ERK signaling is essential for the function of C20orf24 in promoting CRC proliferation. (A) The workflow for identifying the C20orf24-regulated proteins by SILAC-based proteomics. HCT116 cells labeled with "Light chain (Lys 0, Arg 0)" were transfected with flag-C20orf24 plasmid and the cells labeled with "Heavy chain (Lys 8, Arg 10)" were transfected with empty plasmid. The lysates were mixed by 1:1 for quantitation. **(B)** Venn diagram representation of the overlapped proteins identified by three biological

replicates. **(C)** PLGEM model using a regression fitting analysis with a contour plot; black circles showed a good fitting of the DIA-MS data by the PLGEM model. **(D, E)** HCT116 and HT29 cells transfected with C20orf24-overexpressing plasmid were incubated with 250 nM pimasertib or DMSO, and colony formation **(D)** and WST-1 assays **(E)** were performed. Scale bar, 5 mm. **(F)** Western blotting analysis of p-MEK and p-ERK expressions in HCT116 and HT29 cells with indicated treatment. The expression of p-MEK and p-ERK were quantified, and represented as mean ± SD. n.s, non-significant; *, $P < 0.05$; ***, $P < 0.01$.

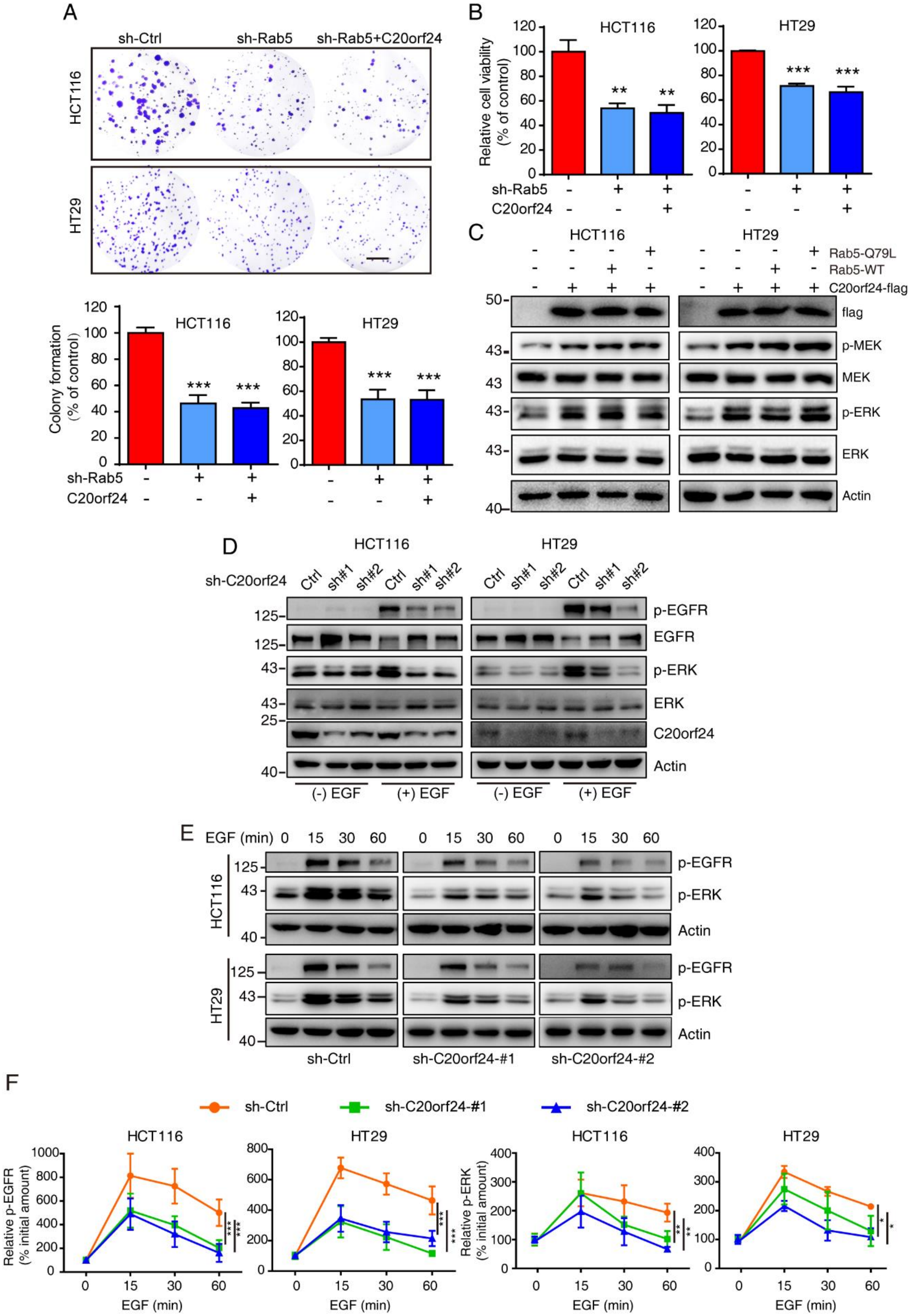
A
sh-Ctrl
sh-Rab5
sh-Rab5+C20orf24
HCT116
HT29
Colony formation (% of control)
sh-Rab5
C20orf24
B
Relative cell viability (% of control)
C
Rab5-Q79L
Rab5-WT
C20orf24-flag
flag
p-MEK
MEK
p-ERK
ERK
Actin
D
sh-C20orf24
Ctrl
sh#1
sh#2
p-EGFR
EGFR
C20orf24
(-) EGF
(+) EGF
E
EGF (min)
sh-Ctrl
sh-C20orf24-#1
sh-C20orf24-#2
F
Relative p-EGFR (% initial amount)
Relative p-ERK (% initial amount)
EGF (min)

Figure S4

Figure S4. Rab5 is the downstream effector for C20orf24-mediated MEK/ERK signaling. (A, B) Colony formation assay and WST-1 assay were performed to determine the abilities to form colonies **(A)** and proliferate **(B)** and in HCT116 and HT29 cells with expressing control or sh-Rab5 with or without C20orf24; Scale bar, 5 mm. (C) C20orf24-overexpressing plasmids were transfected into HCT116 and HT29 cells together with the plasmid expressing Rab5-WT or Rab5-Q79L as indicated; additionally, the expression levels of p-MEK, MEK, p-ERK and ERK were detected by Western blotting. **(D)** C20orf24-depleted HCT116 and HT29 cells in the presence of EGF stimulation were determined for the protein expressions of p-EGFR, EGFR, p-ERK and ERK by immunoblotting. **(E, F)** HCT116 and HT29 cells with knockdown of C20orf24, as well as the control cells, were treated with EGF for up to 60 min, and expression levels of p-EGFR, and p-ERK were determined by Western blotting **(E)**, the bands were subjected to densitometric and statistical analyses **(F)**, comparing all lanes with 0 min for each condition. Bars, SD; *, $P < 0.05$; **, $P < 0.01$; ***, $P < 0.001$.

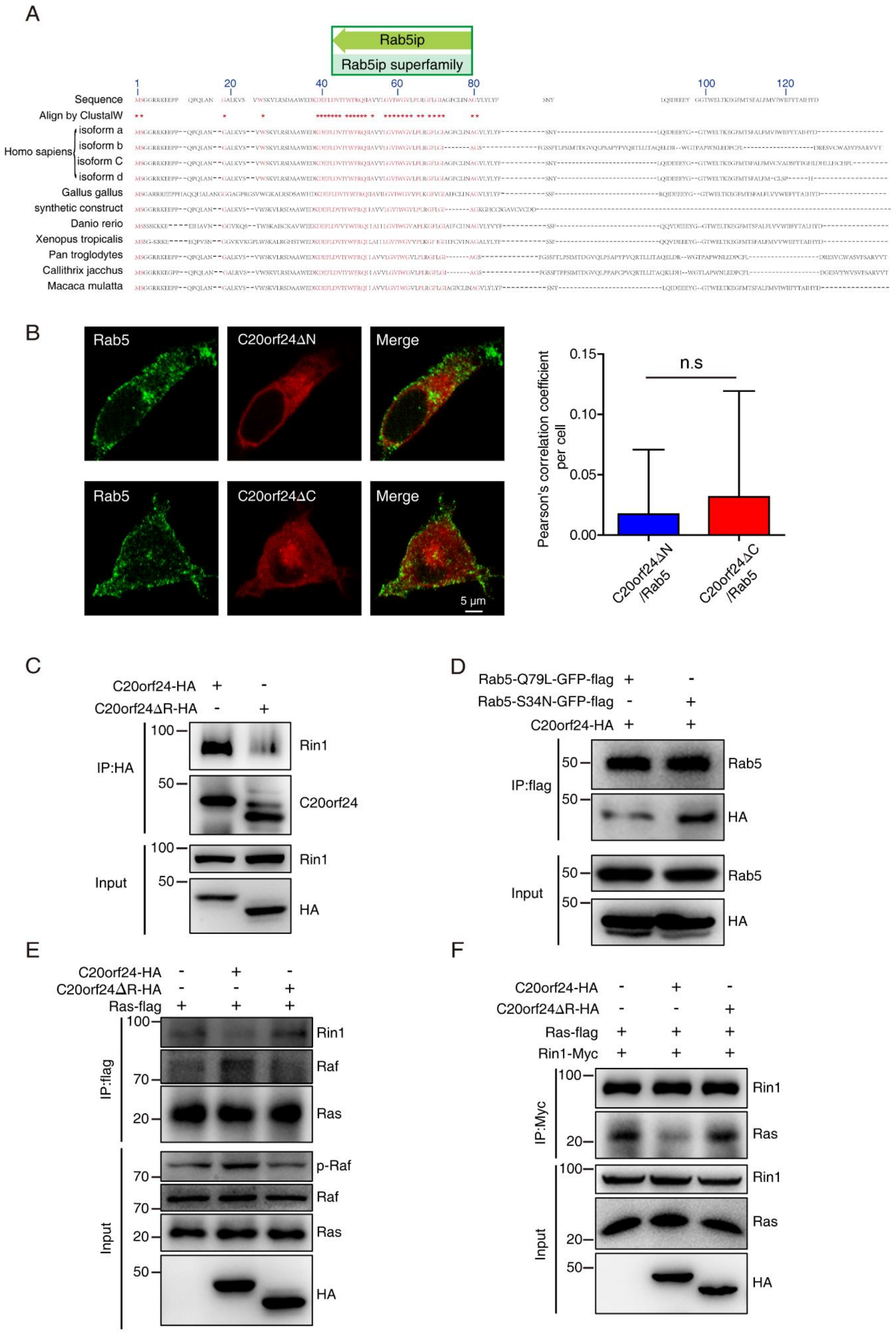
A
Rab5ip
Rab5ip superfamily
Sequence
Align by ClustalW
Homo sapiens
isoform a
isoform b
isoform C
isoform d
Gallus gallus
synthetic construct
Danio rerio
Xenopus tropicalis
Pan troglodytes
Callithrix jacchus
Macaca mulatta
B
Rab5
C20orf24ΔN
Merge
C20orf24ΔC
5 μm
Pearson's correlation coefficient per cell
n.s
C20orf24ΔN /Rab5
C20orf24ΔC /Rab5
C
C20orf24-HA
C20orf24ΔR-HA
IP:HA
Input
Rin1
C20orf24
HA
D
Rab5-Q79L-GFP-flag
Rab5-S34N-GFP-flag
IP:flag
Rab5
E
Ras-flag
Raf
Ras
p-Raf
F
Rin1-Myc
IP:Myc

Figure S5

Figure S5. Rab5ip domain of C20orf24 is required for recruiting Rin1 and activating Ras/Raf signaling. (A) Rab5ip amino acids sequence in different species**.** (B) HCT116 cells expressing mCherry-C20orf24ΔN or mCherry-C20orf24ΔC were subjected to immunostaining for endogenous Rab5, and then imaged by confocal microscopy. The overlaps of Rab5 and C20orf24 (ΔN or ΔC) were quantified. Data are represented as mean ± SD. n = 5-10 cells. ns, non-significant. Scale bar, 5 μm. **(C)** HCT116 cells were transfected with the plasmid expressing C20orf24^{WT} or C20orf24ΔR, and Rin1 expression was detected by Western blotting in the C20orf24 immunoprecipates. **(D)** The binding of C20orf24 to Rab5-Q79L or Rab5-S34N was detected by co-immunoprecipitation assay in HCT116 cells. **(E)** HCT116 cells expressing Ras-flag were transfected with C20orf24^{WT} or C20orf24ΔR plasmid, the lysates were immunoprecipitated with flag antibody, and Western blotting was used to detect the expression levels of Rin1 and Raf. **(F)** HCT116 cells expressing Ras-flag and Rin1-myc were transfected with C20orf24^{WT} or C20orf24ΔR plasmid, and the release of Ras from Rin1 were detected by co-immunoprecipitation assay.

Supplementary Tables

Table S1. Correlation between C20orf24 protein expression level and clinicopathological parameters in 99 cases of colorectal cancer.

Variable	n	Low C20orf24	High C20orf24	*P* value
Age (years)#				
≤55	15	9	6	
>55	84	52	32	1.000
Gender#				
Female	40	25	15	
Male	59	36	23	0.881
T-Stage				
2/3	67	42	25	
4	32	19	13	0.751
N-Stage				
N0	51	40	11	
N1	48	21	27	**0.001*** **
M-Stage				
M0	94	60	34	
M1	5	1	4	**0.049***
Pathologic stage				
Stages I & II	69	44	25	
Stages III & IV	30	17	13	0.504

Table S2. Differentially expressed proteins regulated by C20orf24.

Table S3. C20orf24-binding proteins identified by mass spectrometry.

Table S4. Key resource Table.

Table S2. Differentially expressed proteins regulated by C20orf24.

NO.	Accession	Gene name	Fold change C20orf24/Control	p-value
1	P78330	PSPH	1.97	0.00
2	Q8WTT2	NOC3L	1.56	0.00
3	P24941	CDK2	1.73	0.00
4	P49588	AARS	1.53	0.00
5	P60033	CD81	1.92	0.00
6	Q8TD30	GPT2	1.84	0.00
7	P41250	GARS	1.68	0.00
8	Q9NVP2	ASF1B	1.91	0.00
9	P49327	FASN	1.79	0.00
10	Q14331	FRG1	1.63	0.00
11	P16144	ITGB4	1.78	0.00
12	P53396	ACLY	1.52	0.00
13	P54577	YARS	1.58	0.00
14	Q9UBM7	DHCR7	1.99	0.00
15	Q8ND24	RNF214	1.65	0.00
16	O43175	PHGDH	1.89	0.00
17	Q9BVJ6	UTP14A	2.23	0.00
18	Q8WW12	PCNP	1.53	0.00
19	Q9UHI6	DDX20	1.66	0.00
20	Q12907	LMAN2	2.11	0.00
21	O95453	PARN	1.94	0.00
22	P06756	ITGAV	2.05	0.00
23	Q13427	PPIG	1.64	0.00
24	P14735	IDE	1.69	0.00
25	P07108	DBI	1.63	0.00
26	P08758	ANXA5	1.54	0.00
27	Q14978	NOLC1	2.32	0.00
28	Q9ULT8	HECTD1	1.52	0.00
29	Q53FT3	HIKESHI	1.58	0.00
30	P25685	DNAJB1	1.57	0.00
31	P08238	HSP90AB1	1.66	0.00
32	P22223	CDH3	8.26	0.00
33	P16070	CD44	1.87	0.00
34	P52701	MSH6	1.60	0.00
35	Q92793	CREBBP	1.82	0.00
36	Q9H0A0	NAT10	1.51	0.00
37	Q13418	ILK	1.58	0.00
38	Q14318	FKBP8	1.58	0.00
39	P08195	SLC3A2	2.06	0.00
40	Q9NR19	ACSS2	1.54	0.00
41	Q6RFH5	WDR74	1.77	0.00
42	P35998	PSMC2	1.51	0.00
43	P50579	METAP2	1.54	0.00
44	O95297	MPZL1	2.23	0.00

45	P13473	LAMP2	2.34	0.01
46	Q8WVX9	FAR1	3.72	0.01
47	O60343	TBC1D4	2.23	0.01
48	Q01650	SLC7A5	2.01	0.01
49	P49366	DHPS	1.55	0.01
50	O95573	ACSL3	1.92	0.01
51	Q9Y320	TMX2	2.30	0.01
52	P62805	HIST1H4A	1.60	0.01
53	Q9H5V8	CDCP1	1.70	0.01
54	Q13131	PRKAA1	2.12	0.01
55	P41223	BUD31	2.12	0.01
56	Q8NEZ5	FBXO22	2.02	0.01
57	P46379	BAG6	1.87	0.01
58	P68431	HIST1H3A	1.56	0.01
59	Q15654	TRIP6	1.99	0.01
60	O43665	RGS10	1.68	0.01
61	Q6PGP7	TTC37	1.61	0.01
62	Q9NR09	BIRC6	1.52	0.01
63	Q9H223	EHD4	1.66	0.01
64	O60488	ACSL4	2.39	0.01
65	P35527	KRT9	13.81	0.01
66	Q96S55	WRNIP1	1.86	0.01
67	Q99447	PCYT2	1.58	0.01
68	Q6NZY4	ZCCHC8	1.64	0.01
69	Q13057	COASY	1.67	0.01
70	P52926	HMGA2	1.54	0.01
71	Q9GZQ3	COMMD5	2.19	0.01
72	P43246	MSH2	1.58	0.01
73	Q13153	PAK1	1.82	0.01
74	Q8NBJ4	GOLM1	1.57	0.01
75	Q7Z739	YTHDF3	2.00	0.01
76	O75122	CLASP2	1.53	0.01
77	Q92974	ARHGEF2	1.81	0.01
78	Q9H6T3	RPAP3	1.90	0.01
79	Q03426	MVK	2.40	0.02
80	Q9BY44	EIF2A	1.64	0.02
81	Q2VPK5	CTU2	1.89	0.02
82	P02786	TFRC	1.70	0.02
83	Q12788	TBL3	1.85	0.02
84	Q6Y7W6	GIGYF2	1.52	0.02
85	P04818	TYMS	2.02	0.02
86	O43663	PRC1	1.85	0.02
87	Q13907	IDI1	1.78	0.02
88	Q02952	AKAP12	1.57	0.02
89	Q9BRT6	LLPH	1.92	0.02
90	Q15418	RPS6KA1	2.26	0.02
91	Q14126	DSG2	2.11	0.02
92	Q8WTV0	SCARB1	2.07	0.02

93	Q68CQ4	DIEXF	1.50	0.02
94	P04792	HSPB1	1.62	0.02
95	Q6KC79	NIPBL	1.85	0.02
96	Q12768	WASHC5	1.71	0.02
97	Q8IUI8	CRLF3	1.66	0.02
98	Q7Z5K2	WAPL	1.51	0.02
99	Q13085	ACACA	1.70	0.02
100	O75367	H2AFY	1.53	0.02
101	P23229	ITGA6	2.05	0.02
102	Q9NQH7	XPNPEP3	2.57	0.02
103	O75391	SPAG7	1.62	0.02
104	Q5BJH7	YIF1B	1.60	0.02
105	Q9Y617	PSAT1	1.92	0.02
106	Q6PIU2	NCEH1	1.57	0.02
107	P51812	RPS6KA3	1.95	0.02
108	P29218	IMPA1	1.82	0.02
109	P20339	RAB5A	1.55	0.02
110	O43847	NRDC	1.69	0.02
111	Q96P48	ARAP1	2.31	0.02
112	O75475	PSIP1	2.17	0.03
113	O60749	SNX2	1.56	0.03
114	Q9UEW8	STK39	1.76	0.03
115	Q8N122	RPTOR	1.67	0.03
116	P46976	GYG1	2.20	0.03
117	Q8NBJ5	COLGALT1	1.53	0.03
118	Q6XZF7	DNMBP	1.57	0.03
119	Q16881	TXNRD1	1.68	0.03
120	Q5UCC4	EMC10	1.75	0.03
121	Q8WUX1	SLC38A5	3.07	0.03
122	O94973	AP2A2	1.56	0.03
123	Q6P1N9	TATDN1	1.55	0.03
124	O15446	CD3EAP	1.85	0.03
125	P08243	ASNS	2.36	0.03
126	Q9Y6N5	SQRDL	1.88	0.03
127	O00767	SCD	2.92	0.03
128	P21583	KITLG	1.79	0.03
129	O75792	RNASEH2A	2.22	0.04
130	P39656	DDOST	1.72	0.04
131	Q96AC1	FERMT2	2.29	0.04
132	Q9BXY0	MAK16	1.67	0.04
133	Q96HW7	INTS4	1.57	0.04
134	Q9UET6	FTSJ1	2.03	0.04
135	P04264	KRT1	11.64	0.04
136	P83436	COG7	1.98	0.04
137	P62851	RPS25	1.71	0.04
138	O75821	EIF3G	1.59	0.04
139	P05386	RPLP1	1.82	0.04
140	O15231	ZNF185	3.04	0.04

141	Q9Y624	F11R	1.83	0.04
142	P31153	MAT2A	1.71	0.04
143	Q16222	UAP1	1.89	0.04
144	Q8TBB5	KLHDC4	1.63	0.04
145	Q01581	HMGCS1	2.68	0.05
146	Q7Z7F7	MRPL55	1.58	0.05
147	Q52LW3	ARHGAP29	4.08	0.05
148	Q8N5K1	CISD2	1.61	0.05
149	Q96SB4	SRPK1	1.61	0.05
150	O43657	TSPAN6	1.60	0.05
151	P01130	LDLR	2.60	0.05
152	O15037	KHNYN	-1.64	0.00
153	O15305	PMM2	-1.53	0.00
154	P00558	PGK1	-1.60	0.00
155	Q9Y4C1	KDM3A	-1.98	0.00
156	P04075	ALDOA	-1.52	0.00
157	O15479	MAGEB2	-1.50	0.00
158	P31947	SFN	-1.52	0.00
159	P05783	KRT18	-1.89	0.00
160	P12532	CKMT1A	-1.57	0.00
161	P56182	RRP1	-1.72	0.00
162	O95786	DDX58	-2.29	0.00
163	Q93084	ATP2A3	-4.02	0.00
164	Q6NW34	NEPRO	-1.68	0.00
165	Q9Y2Q3	GSTK1	-1.70	0.00
166	P54819	AK2	-1.56	0.00
167	P56556	NDUFA6	-1.55	0.00
168	Q9BPW8	NIPSNAP1	-1.53	0.00
169	P13674	P4HA1	-2.35	0.00
170	Q13510	ASAH1	-1.77	0.00
171	Q08AF3	SLFN5	-2.23	0.00
172	Q9P0M6	H2AFY2	-1.85	0.00
173	P49642	PRIM1	-1.94	0.00
174	P05787	KRT8	-1.85	0.00
175	Q02978	SLC25A11	-1.62	0.00
176	P42224	STAT1	-6.59	0.00
177	Q13084	MRPL28	-2.40	0.00
178	Q16698	DECR1	-1.67	0.00
179	Q9BW91	NUDT9	-2.53	0.00
180	Q9H7D0	DOCK5	-1.59	0.00
181	Q56VL3	OCIAD2	-1.86	0.00
182	Q9Y333	LSM2	-1.83	0.00
183	P49748	ACADVL	-1.96	0.00
184	Q8IYS2	KIAA2013	-1.79	0.00
185	O76024	WFS1	-1.63	0.00
186	Q9UDT6	CLIP2	-2.14	0.00
187	P27338	MAOB	-1.60	0.00
188	Q13601	KRR1	-1.84	0.00

189	P06744	GPI	-1.65	0.00
190	Q9UJS0	SLC25A13	-1.73	0.00
191	O43676	NDUFB3	-1.75	0.00
192	Q9H9P8	L2HGDH	-1.82	0.00
193	Q8N5M1	ATPAF2	-1.75	0.00
194	P40121	CAPG	-1.61	0.00
195	Q9BUT1	BDH2	-2.52	0.00
196	P32320	CDA	-2.72	0.00
197	Q969Z3	43892	-2.13	0.00
198	P09972	ALDOC	-1.84	0.01
199	Q96FJ2	DYNLL2	-1.68	0.01
200	Q8N543	OGFOD1	-1.78	0.01
201	Q9P2N5	RBM27	-1.66	0.01
202	Q96HC4	PDLIM5	-1.56	0.01
203	Q8NFV4	ABHD11	-1.78	0.01
204	Q14155	ARHGEF7	-2.66	0.01
205	P49770	EIF2B2	-1.88	0.01
206	P21281	ATP6V1B2	-1.57	0.01
207	Q9BRJ2	MRPL45	-1.52	0.01
208	Q9H9A6	LRRC40	-1.76	0.01
209	Q8IUX4	APOBEC3F	-2.13	0.01
210	Q03519	TAP2	-2.00	0.01
211	Q9BX40	LSM14B	-1.53	0.01
212	Q9NUJ1	ABHD10	-1.73	0.01
213	P14854	COX6B1	-1.52	0.01
214	P52815	MRPL12	-1.54	0.01
215	O00116	AGPS	-1.67	0.01
216	P07305	H1F0	-1.91	0.01
217	Q7Z406	MYH14	-2.10	0.01
218	P05161	ISG15	-1.78	0.01
219	Q53H12	AGK	-1.53	0.01
220	O00425	IGF2BP3	-1.53	0.01
221	Q9H7L9	SUDS3	-1.58	0.01
222	Q8WXA9	SREK1	-1.57	0.01
223	Q00169	PITPNA	-1.69	0.01
224	Q8WXF1	PSPC1	-1.54	0.01
225	Q9Y6K5	OAS3	-2.02	0.01
226	P30740	SERPINB1	-2.09	0.01
227	Q99536	VAT1	-1.86	0.01
228	P04183	TK1	-1.96	0.01
229	O00762	UBE2C	-1.81	0.01
230	Q9NR45	NANS	-1.54	0.01
231	Q9UHY7	ENOPH1	-2.21	0.01
232	P00966	ASS1	-3.64	0.01
233	Q8NFW8	CMAS	-2.20	0.01
234	Q6ZXV5	TMTC3	-2.20	0.01
235	Q9Y394	DHRS7	-1.67	0.01
236	P63173	RPL38	-1.53	0.01

237	Q8TAQ2	SMARCC2	-1.68	0.01
238	Q01085	TIAL1	-1.87	0.01
239	P55795	HNRNPH2	-1.61	0.01
240	P34896	SHMT1	-1.54	0.01
241	Q99538	LGMN	-2.73	0.01
242	Q9Y3Z3	SAMHD1	-1.83	0.02
243	Q9UNF0	PACSIN2	-1.50	0.02
244	Q16762	TST	-3.34	0.02
245	P47895	ALDH1A3	-2.12	0.02
246	P42695	NCAPD3	-1.73	0.02
247	P06396	GSN	-2.34	0.02
248	O94808	GFPT2	-3.20	0.02
249	Q16836	HADH	-1.97	0.02
250	Q9H074	PAIP1	-1.60	0.02
251	Q96A65	EXOC4	-1.53	0.02
252	Q6UXV4	APOOL	-4.28	0.02
253	P09455	RBP1	-1.93	0.02
254	P11234	RALB	-1.61	0.02
255	Q13868	EXOSC2	-1.64	0.02
256	Q9H7D7	WDR26	-1.65	0.02
257	P12109	COL6A1	-2.89	0.02
258	Q6UX53	METTL7B	-1.93	0.02
259	P48735	IDH2	-1.95	0.02
260	P62487	POLR2G	-1.86	0.02
261	Q7Z7N9	TMEM179B	-1.74	0.02
262	O95400	CD2BP2	-1.78	0.02
263	Q5VZ89	DENND4C	-1.76	0.02
264	Q02252	ALDH6A1	-1.95	0.02
265	P29373	CRABP2	-2.92	0.02
266	Q969L2	MAL2	-1.74	0.02
267	Q14914	PTGR1	-2.48	0.02
268	P37837	TALDO1	-1.68	0.02
269	Q6NUK1	SLC25A24	-1.88	0.02
270	P30085	CMPK1	-1.60	0.03
271	Q9H4G0	EPB41L1	-1.60	0.03
272	O95210	STBD1	-3.80	0.03
273	Q9BQ39	DDX50	-2.17	0.03
274	P49590	HARS2	-1.96	0.03
275	P55084	HADHB	-2.04	0.03
276	Q8TB61	SLC35B2	-1.94	0.03
277	P09104	ENO2	-1.97	0.03
278	P40818	USP8	-1.53	0.03
279	Q9NXA8	SIRT5	-1.52	0.03
280	P10321	HLA-C	-2.37	0.03
281	P07602	PSAP	-1.83	0.03
282	P52735	VAV2	-1.56	0.03
283	Q9Y6Q5	AP1M2	-2.35	0.03
284	P99999	CYCS	-1.65	0.03

285	Q99720	SIGMAR1	-1.64	0.03
286	Q92817	EVPL	-1.73	0.03
287	Q9HCY8	S100A14	-2.95	0.03
288	Q15370	ELOB	-2.13	0.03
289	P19367	HK1	-2.14	0.03
290	Q96A26	FAM162A	-1.66	0.03
291	P31040	SDHA	-1.60	0.03
292	Q86X55	CARM1	-1.56	0.03
293	Q9UKF6	CPSF3	-1.86	0.03
294	Q96RQ3	MCCC1	-4.19	0.04
295	O94905	ERLIN2	-2.16	0.04
296	Q14554	PDIA5	-1.53	0.04
297	P04066	FUCA1	-3.52	0.04
298	Q75QN2	INTS8	-1.62	0.04
299	Q9Y5Q8	GTF3C5	-2.28	0.04
300	P04424	ASL	-1.75	0.04
301	O94888	UBXN7	-1.69	0.04
302	Q96JM3	CHAMP1	-1.60	0.04
303	Q96CN7	ISOC1	-2.35	0.04
304	Q9BYD2	MRPL9	-1.73	0.04
305	Q96GG9	DCUN1D1	-1.59	0.04
306	P30042	C21orf33	-1.89	0.04
307	Q9NWU1	OXSM	-2.01	0.04
308	Q14195	DPYSL3	-3.69	0.04
309	Q86TI2	DPP9	-1.68	0.04
310	Q8WZA0	LZIC	-1.66	0.04
311	Q8IZP0	ABI1	-2.10	0.04
312	O95372	LYPLA2	-1.89	0.05
313	P39687	ANP32A	-1.52	0.05
314	Q96JB2	COG3	-1.69	0.05

Table S3. C20orf24 binding proteins identified by mass spectrometry

NO.	Accession	Gene name	Description	MW [kDa]	PEP Score	Coverage	Peptides
1	P20339	RAB5A	Ras-related protein Rab-5A	23.6	132.46	70.70	14
2	P60660	MYL6	Myosin light polypeptide 6	16.9	72.26	66.89	11
3	P16083	NQO2	Ribosyldihydronicotinamide dehydrogenase	25.9	46.65	44.59	10
4	P62269	RPS18	40S ribosomal protein S18	17.7	22.50	43.42	10
5	O14950	MYL12B	Myosin regulatory light chain 12B	19.8	43.19	51.16	9
6	P60709	ACTB	Actin, cytoplasmic 1	41.7	17.41	22.13	8
7	P61254	RPL26	60S ribosomal protein L26	17.2	15.07	33.79	8
8	O15145	ARPC3	Actin-related protein 2/3 complex subunit 3	20.5	22.16	34.83	7
9	Q06830	PRDX1	Peroxiredoxin-1	22.1	9.80	36.18	6
10	P24844	MYL9	Myosin regulatory light polypeptide 9	19.8	29.46	44.19	6
11	P83731	RPL24	60S ribosomal protein L24	17.8	12.00	35.67	6
12	P62750	RPL23A	60S ribosomal protein L23a	17.7	10.25	32.69	6
13	P51148	RAB5C	Ras-related protein Rab-5C	23.5	22.72	27.31	5
14	P59998	ARPC4	Actin-related protein 2/3 complex subunit 4	19.7	11.05	27.98	5
15	P46779	RPL28	60S ribosomal protein L28	15.7	6.23	33.58	5
16	P62249	RPS16	40S ribosomal protein S16	16.4	10.48	35.62	5
17	P02768	ALB	Serum albumin	69.3	17.82	6.08	4
18	P62937	PPIA	Peptidyl-prolyl cis-trans isomerase A	18	8.12	24.85	4
19	P22392	NME2	Nucleoside diphosphate kinase B	17.3	8.86	31.58	4
20	P15531	NME1	Nucleoside diphosphate kinase A	17.1	10.65	34.21	4
21	P35579	MYH9	Myosin-9	226.4	12.22	2.70	4
22	O60814	HIST1H2BK	Histone H2B type 1-K	13.9	11.97	34.92	4
23	P06899	HIST1H2BJ	Histone H2B type 1-J	13.9	12.05	34.92	4
24	P0C0S8	HIST1H2AG	Histone H2A type 1	14.1	6.69	35.38	4
25	P62158	CALM1	Calmodulin	16.8	15.86	30.87	4
26	Q9BPX5	ARPC5L	Actin-related protein 2/3 complex subunit 5-like protein	16.9	13.48	33.99	4
27	O15511	ARPC5	Actin-related protein 2/3 complex subunit 5	16.3	10.03	34.44	4
28	P62829	RPL23	60S ribosomal protein L23	14.9	7.57	36.43	4
29	Q02543	RPL18A	60S ribosomal protein L18a	20.7	6.66	23.86	4
30	P30050	RPL12	60S ribosomal protein L12	17.8	12.96	33.94	4

31	P62913	RPL11	60S ribosomal protein L11	20.2	6.76	21.35	4
32	P62277	RPS13	40S ribosomal protein S13	17.2	10.56	28.48	4
33	P62280	RPS11	40S ribosomal protein S11	18.4	4.80	23.42	4
34	P32119	PRDX2	Peroxiredoxin-2	21.9	3.43	13.13	3
35	P16402	HIST1H1D	Histone H1.3	22.3	7.85	10.86	3
36	O00422	SAP18	Histone deacetylase complex subunit SAP18	17.6	4.87	20.26	3
37	P23528	CFL1	Cofilin-1	18.5	7.85	19.88	3
38	Q8IX12	CCAR1	Cell division cycle and apoptosis regulator protein 1	132.7	6.83	3.39	3
39	P49207	RPL34	60S ribosomal protein L34	13.3	3.75	14.53	3
40	P46776	RPL27A	60S ribosomal protein L27a	16.6	4.35	23.65	3
41	P61353	RPL27	60S ribosomal protein L27	15.8	6.60	22.06	3
42	P18621	RPL17	60S ribosomal protein L17	21.4	3.70	14.67	3
43	P05387	RPLP2	60S acidic ribosomal protein P2	11.7	4.50	55.65	3
44	P62851	RPS25	40S ribosomal protein S25	13.7	3.56	20.80	3
45	Q9BUV8	C20orf24	Uncharacterized protein RAB5IF	15.5	13.96	11.68	2
46	P07477	PRSS1	Trypsin-1	26.5	2.91	12.15	2
47	Q9BVC6	TMEM109	Transmembrane protein 109	26.2	1.82	8.64	2
48	Q9Y2W1	THRAP3	Thyroid hormone receptor-associated protein 3	108.6	6.27	2.83	2
49	P63208	SKP1	S-phase kinase-associated protein 1	18.6	3.09	11.66	2
50	Q04837	SSBP1	Single-stranded DNA-binding protein, mitochondrial	17.2	4.14	15.54	2
51	P62820	RAB1A	Ras-related protein Rab-1A	22.7	4.22	13.17	2
52	P46940	IQGAP1	Ras GTPase-activating-like protein IQGAP1	189.1	2.53	1.33	2
53	P30086	PEBP1	Phosphatidylethanolamine-binding protein 1	21	4.61	14.97	2
54	P23284	PPIB	Peptidyl-prolyl cis-trans isomerase B	23.7	3.12	9.72	2
55	P62805	HIST1H4A	Histone H4	11.4	2.63	21.36	2
56	P68431	HIST1H3A	Histone H3.1	15.4	2.47	14.71	2
57	P17096	HMGA1	High mobility group protein HMG-I/HMG-Y	11.7	8.48	23.36	2
58	P22626	HNRNPA2B1	Heterogeneous nuclear ribonucleoproteins A2/B1	37.4	2.15	7.08	2
59	P09651	HNRNPA1	Heterogeneous nuclear ribonucleoprotein A1	38.7	2.62	6.72	2
60	P62633	CNBP	Cellular nucleic acid-binding protein	19.5	3.13	15.25	2
61	P13987	CD59	CD59 glycoprotein	14.2	8.48	18.75	2
62	Q9NYF8	BCLAF1	Bcl-2-associated transcription factor 1	106.1	5.28	3.04	2
63	P07741	APRT	Adenine phosphoribosyltransferase	19.6	2.20	12.78	2

64	P42766	RPL35	60S ribosomal protein L35	14.5	4.81	15.45	2
65	P62899	RPL31	60S ribosomal protein L31	14.5	4.11	17.60	2
66	P35268	RPL22	60S ribosomal protein L22	14.8	3.26	18.75	2
67	Q07020	RPL18	60S ribosomal protein L18	21.6	3.72	12.77	2
68	P62854	RPS26	40S ribosomal protein S26	13	4.30	20.87	2
69	P60866	RPS20	40S ribosomal protein S20	13.4	10.83	19.33	2
70	P08708	RPS17	40S ribosomal protein S17	15.5	11.34	15.56	2
71	P62263	RPS14	40S ribosomal protein S14	16.3	4.17	9.27	2
72	P13010	XRCC5	X-ray repair cross-complementing protein 5	82.7	2.20	1.91	1
73	P68363	TUBA1B	Tubulin alpha-1B chain	50.1	1.11	2.22	1
74	P35030	PRSS3	Trypsin-3	32.5	2.24	4.28	1
75	P37802	TAGLN2	Transgelin-2	22.4	2.27	6.03	1
76	P00441	SOD1	Superoxide dismutase	15.9	1.55	9.09	1
77	P38646	HSPA9	Stress-70 protein, mitochondrial	73.6	0.93	1.33	1
78	Q9H169	STMN4	Stathmin-4	22.1	2.41	4.23	1
79	Q14247	CTTN	Src substrate cortactin	61.5	1.02	2.36	1
80	P55854	SUMO3	Small ubiquitin-related modifier 3	11.6	1.56	11.65	1
81	P09132	SRP19	Signal recognition particle 19 kDa protein	16.1	1.13	10.42	1
82	Q5BJF2	TMEM97	Sigma intracellular receptor 2	20.8	1.00	4.55	1
83	Q9Y3S1	WNK2	Serine/threonine-protein kinase WNK2	242.5	0.82	0.78	1
84	Q16629	SRSF7	Serine/arginine-rich splicing factor 7	27.4	1.22	3.78	1
85	P00734	F2	Prothrombin	70	1.02	2.41	1
86	P28072	PSMB6	Proteasome subunit beta type-6	25.3	0.89	4.18	1
87	Q9GZZ1	NAA50	N-alpha-acetyltransferase 50	19.4	1.57	6.51	1
88	O75251	NDUFS7	NADH dehydrogenase iron-sulfur protein 7	23.5	0.86	4.23	1
89	P35580	MYH10	Myosin-10	228.9	3.71	0.76	1
90	Q9UBX7	KLK11	Kallikrein-11	31	0.93	2.48	1
91	Q86YZ3	HRNR	Hornerin	282.2	3.71	1.33	1
92	O15347	HMGB3	High mobility group protein B3	23	2.43	6.50	1
93	Q9NR31	SAR1A	GTP-binding protein SAR1a	22.4	1.79	5.56	1
94	P28799	GRN	Granulins	63.5	0.89	1.35	1
95	P26641	EEF1G	Elongation factor 1-gamma	50.1	0.84	1.60	1
96	P62487	POLR2G	DNA-directed RNA polymerase II subunit RPB7	19.3	1.93	7.56	1

97	P00403	MT-CO2	Cytochrome c oxidase subunit 2	25.5	1.00	4.41	1
98	Q9H8M2	BRD9	Bromodomain-containing protein 9	67	1.32	1.17	1
99	P48047	ATP5O	ATP synthase subunit O, mitochondrial	23.3	2.21	5.16	1
100	P53999	SUB1	Activated RNA polymerase II transcriptional coactivator p15	14.4	2.33	10.24	1
101	Q02878	RPL6	60S ribosomal protein L6	32.7	1.72	3.82	1
102	Q9Y3U8	RPL36	60S ribosomal protein L36	12.2	1.19	8.57	1
103	P62910	RPL32	60S ribosomal protein L32	15.9	2.27	10.37	1
104	P62888	RPL30	60S ribosomal protein L30	12.8	0.96	10.43	1
105	P26373	RPL13	60S ribosomal protein L13	24.2	0.93	5.21	1
106	P05386	RPLP1	60S acidic ribosomal protein P1	11.5	3.38	14.04	1
107	P62241	RPS8	40S ribosomal protein S8	24.2	1.87	5.29	1
108	P62081	RPS7	40S ribosomal protein S7	22.1	2.43	4.12	1
109	P62753	RPS6	40S ribosomal protein S6	28.7	1.79	4.82	1
110	P46782	RPS5	40S ribosomal protein S5	22.9	2.23	7.35	1
111	P62847	RPS24	40S ribosomal protein S24	15.4	2.41	11.28	1
112	P62266	RPS23	40S ribosomal protein S23	15.8	1.53	5.59	1
113	P39019	RPS19	40S ribosomal protein S19	16.1	1.28	6.90	1
114	P62244	RPS15A	40S ribosomal protein S15a	14.8	1.71	6.92	1
115	P62841	RPS15	40S ribosomal protein S15	17	5.61	8.28	1
116	P46783	RPS10	40S ribosomal protein S10	18.9	2.10	5.45	1
117	Q9NRX2	MRPL17	39S ribosomal protein L17	20	0.95	4.57	1

Table S4: KEY RESOURCES TABLE

REAGENT or RESOURCE	SOURCE	IDENTIFIER
**Antibodies**		
Rabbit Polyclonal anti-C20orf24	Sigma	Cat#SAB2108059
Rabbit Polyclonal anti-C20orf24	Biorbyt	Cat#orb155989
Rabbit Polyclonal anti-C20orf24	Sino Biological	Cat#202591-T10
Rabbit Polyclonal anti-EGFR	Proteintech	Cat#18986-1-AP
Mouse Monoclonal anti-β-actin	Proteintech	Cat#60008-1-Ig
Rabbit Polyclonal anti-Rab5A	Proteintech	Cat#11947-1-AP
Rabbit Polyclonal anti-HA	Proteintech	Cat#51064-2-AP
Rabbit Polyclonal anti-flag	Proteintech	Cat#20543-1-AP
Rabbit Polyclonal anti-GST	Proteintech	Cat#10000-0-AP
Rabbit Polyclonal anti-Rin1	Proteintech	Cat#16388-1-AP
Rabbit Polyclonal anti-Hras	Proteintech	Cat#18295-1-AP
Mouse Monoclonal anti-Flag	MBL	Cat#M185-3L
anti-Phospho-EGFR-Y1086 pAb	Abclonal	Cat#AP0301
anti-Phospho-ERK1-T202/Y204+ERK2-T185/Y187 pAb	Abclonal	Cat#AP0472
anti-Phospho-MEK1 (Thr286) Antibody	CST	Cat#9127
anti-Phospho-c-Raf (Ser338) Antibody	CST	Cat#9427
Mouse Monoclonal anti-MEK1	CST	Cat#2352
Mouse Monoclonal anti-E-Cadherin	CST	Cat#14472
Rabbit Monoclonal anti-Vimentin	CST	Cat#5741
Rabbit Monoclonal anti-β-Catenin	CST	Cat#8480
**Biological Samples**		
CRC tissue microarray chip	Shanghai OUTDO Biotech Co.	Cat#HCol-Ade180Sur-06
**Chemicals and Purified Proteins**		
Recombinant Human EGF	PEPROTECH	Cat#AF-100-15
Pimasertib	Selleck	Cat#AS-703026
Protein A/G PLUS-Agarose	Santa Cruz	Cat#sc-2003
GST-R5BD	This paper	N/A
**Commercial Assays**		
PrimeScript RT reagent Kit with gDNA Eraser	Takara	Cat#DRR047A
QuikChange Site-Directed Mutagenesis Kit	Agilent Technologies	Cat#200518

REAGENT or RESOURCE		
Experimental Models: Cell Lines		
Human: HTC-116	ATCC	Cat#CCL-247; RRID: CVCL_0291
Human: HT29	ATCC	Cat#HTB-38
Human: HEK293T	ATCC	Cat#CRL-11268; RRID: CVCL_1926
**Animal Models:**		
Mouse: Male BALB/c nu (3-4 weeks old)	GemPharmatech Co., Ltd	N/A
**Oligonucleotides**		
siC20orf24 #1：5’-CGUGUUUCCUGGACCGCGATT-3’	GenePharma	N/A
siC20orf24 #2：5’-CGUGGUCCAUAGCACAGUATT-3’	GenePharma	
**Primers for qRT-PCR**		
C20orf24-UP：5’-GAGTAAGGTGCTGCGGAG-3’	Ruibiotech, Guangzhou, China	
C20orf24-Down：5’-AAATGACCATGAACAAGGC-3’	Ruibiotech, Guangzhou, China	
TGIF2-UP: 5’-TTCGGAAGGATGGCAAAGACCC-3’	Ruibiotech, Guangzhou, China	
TGIF2-Down: 5’-CTGAGTGAAGCGGCATGGAGCA-3’	Ruibiotech, Guangzhou, China	
TGIF2-C20orf24-UP: 5’-CCTGCCCAAGGAGTCGGTGAAGA-3’	Ruibiotech, Guangzhou, China	
TGIF2-C20orf24-Down: 5’-AAATGACACCCAGGACCACAGCA-3’	Ruibiotech, Guangzhou, China	
ACTB-UP：5’-ACGTGGACATCCGCAAAG-3’	Ruibiotech, Guangzhou, China	
ACTB-Down：5’-GACTCGTCATACTCCTGCTTG-3’	Ruibiotech, Guangzhou, China	
**Recombinant Plasmids**		
pcDNA3.1(+)	Invitrogen	Cat#V790-20
pEGFP-N1 (GFPwt)	Clontech	Cat#6085-1
pcDNA3.1-C20orf24-flag	This paper	N/A
pcDNA3.1-TGIF2-flag	This paper	N/A
pcDNA3.1-TGIF2-C20orf24-flag	This paper	N/A
pLVX-mCherry-HA-C20orf24	This paper	N/A
pLVX-mCherry-HA-C20orf24-ΔR	This paper	N/A
pLVX-mCherry-HA-C20orf24-ΔN	This paper	N/A
pLVX-mCherry-HA-C20orf24-ΔC	This paper	N/A
pLVX-C20orf24-ΔR-HA	This paper	N/A
pLVX-C20orf24-HA	This paper	N/A
pLVX-Rin1-Myc	This paper	N/A
pCMV-Ras-flag	This paper	N/A
pLVX-Rab5-WT-GFP-flag	This paper	N/A

pLVX-Rab5-S34N-GFP-flag	This paper	N/A
pLVX-Rab5-Q79L-GFP-flag	This paper	N/A
Lentivirus pLKO.1-sh-C20orf24-#1	This paper,TranSheepBio	N/A
Lentivirus pLKO.1-sh-C20orf24-#2	This paper,TranSheepBio	N/A
**Software and Algorithms**		
GraphPad Prism 5	N/A	https://www.graphpad.com/scientific-software/prism/
Cytoscape	N/A	http://cytoscape.org/
Proteome Discoverer	Thermo Fisher Scientific	N/A
Ingenuity Pathway Analysis	Ingenuity Systems	N/A
